# Cost of Preventing, Managing, and Treating Human Papillomavirus (HPV)-Related Diseases in Sweden before the Introduction of Quadrivalent HPV Vaccination

**DOI:** 10.1371/journal.pone.0139062

**Published:** 2015-09-23

**Authors:** Ellinor Östensson, Maria Fröberg, Amy Leval, Ann-Cathrin Hellström, Magnus Bäcklund, Niklas Zethraeus, Sonia Andersson

**Affiliations:** 1 Department of Women’s and Children’s Health, Division of Obstetrics and Gynecology, Karolinska University Hospital, Karolinska Institutet, Stockholm, Sweden; 2 Department of Medical Epidemiology and Biostatistics, Karolinska Institutet, Stockholm, Sweden; 3 Department of Neurobiology, Care Science and Society, Centre for Family Medicine and Community Medicine, Karolinska Institutet, Stockholm, Sweden; 4 Department of Medicine, Infectious Disease Unit, Karolinska Institutet, Stockholm, Sweden; 5 Department of Communicable Disease Control and Prevention for Stockholm County, Stockholm, Sweden; 6 Department of Oncology-Pathology, Karolinska University Hospital-Solna, Karolinska Institutet, Stockholm, Sweden; 7 Department of Medicine, Solna (MedS), K2, Karolinska Institutet, Stockholm, Sweden; 8 Medical Management Centre (MMC), Department of Learning, Informatics, Management and Ethics (LIME), Karolinska Institutet, Stockholm, Sweden; State University of Maringá/Universidade Estadual de Maringá, BRAZIL

## Abstract

**Objective:**

Costs associated with HPV-related diseases such as cervical dysplasia, cervical cancer, and genital warts have not been evaluated in Sweden. These costs must be estimated in order to determine the potential savings if these diseases were eradicated and to assess the combined cost-effectiveness of HPV vaccination and cervical cancer screening. The present study aimed to estimate prevention, management, and treatment costs associated with cervical dysplasia, cervical cancer, and genital warts from a societal perspective in Sweden in 2009, 1 year before the quadrivalent HPV vaccination program was implemented.

**Methods and Materials:**

Data from the Swedish cervical cancer screening program was used to calculate the costs associated with prevention (cytological cervical cancer screening), management (colposcopy and biopsy following inadequate/abnormal cytological results), and treatment of CIN. Swedish official statistics were used to estimate treatment costs associated with cervical cancer. Published epidemiological data were used to estimate the number of incident, recurrent, and persistent cases of genital warts; a clinical expert panel assessed management and treatment procedures. Estimated visits, procedures, and use of medications were used to calculate the annual cost associated with genital warts.

**Results:**

From a societal perspective, total estimated costs associated with cervical cancer and genital warts in 2009 were €106.6 million, of which €81.4 million (76%) were direct medical costs. Costs associated with prevention, management, and treatment of CIN were €74 million; screening and management costs for women with normal and inadequate cytology alone accounted for 76% of this sum. The treatment costs associated with incident and prevalent cervical cancer and palliative care were €23 million. Estimated costs for incident, recurrent and persistent cases of genital warts were €9.8 million.

**Conclusion:**

Prevention, management, and treatment costs associated with cervical dysplasia, cervical cancer, and genital warts are substantial. Defining these costs is important for future cost-effectiveness analyses of the quadrivalent HPV vaccination program in Sweden.

## Introduction

Each year 34 300 women in Europe are diagnosed with cervical cancer, and 16 300 die from this disease [1]. In addition, around 700 000 men and women in Europe are diagnosed with genital warts annually [2], and a report from the Nordic countries has shown that 1 in 10 women are diagnosed with genital warts before 45 years of age [3]. Human papillomavirus (HPV) infection is a necessary cause of cervical cancer and genital warts [4,5]. At present, approximately 70% of all cervical cancer cases are attributable to the oncogenic HPV types 16 and 18, and an additional 10% are attributable to the oncogenic HPV types 45 and 31 [6,7]. The non-oncogenic HPV types 6 and 11 cause low-grade cervical lesions and about 90% of all cases of genital warts [8–10]. In contrast to other malignancies, cervical cancer is more prevalent among younger women, with incidence rising rapidly after the age of 25–30 years. However, this incidence could be prevented by adequate screening, and primary prevention with HPV vaccination [11].

The Swedish cervical cancer screening program has been in place since the early 1960s, and has effectively reduced the prevalence of cervical cancer in the country by 50% [12]. However, every year in Sweden around 450 women, approximately 40% of whom are below 45 years of age, are diagnosed with cervical cancer, and around 150 women die from the disease despite the existence of the screening program [13]. The coverage of the Swedish cervical cancer screening program is around 80%, which is lower than that recommended by the European Union, and may contribute to cervical cancer mortality [14]. Also, it has been reported that the Swedish cervical cancer screening program lacks sensitivity and precision to detect cervical intraepithelial neoplasia (CIN) and cervical cancer [15].

High-risk HPV testing, i.e. detection of high-risk HPV DNA, is an effective screening method to prevent cervical cancer. When used in primary cervical cancer screening, samples taken by clinicians show the highest sensitivity to detect CIN [16], though self-collected vaginal samples (samples collected by women in the privacy of their own home), can also be used to improve screening coverage [17]. High-risk-HPV testing is used for triage of low-grade cytological abnormalities in Sweden, but it is not currently used in primary cervical cancer screening. Instead, the Swedish cervical cancer screening program currently utilizes either conventional or liquid-based cytology for primary screening. The guidelines of the Swedish cervical cancer screening program recommend that women 23–60 years of age be screened at 3-year (for women 23–50 years of age) or 5-year (for women 51–60 years of age) intervals. According to recent reports, every year about 3% to 5% of all cytological results show some kind of abnormality, about 80% of which are minor [18].

Genital warts are benign mucosal or skin lesions that are common in sexually active men and women. They are usually located at sites that are exposed to epithelial contact during sexual intercourse, most commonly at external genital sites, but they can also be found at internal sites such as the vagina and the uterine ectocervix in women, and the anus in both men and women. Genital warts are often asymptomatic, but they can be psychologically troublesome, and sometimes cause itching, burning, and dyspareunia. Treatment of genital warts depends on several clinical aspects, such as patient preference, planned or known pregnancy, and location and extent of the lesions. Since genital warts are benign and sometimes regress spontaneously, a wait-and-see approach can sometimes be applied. In Sweden, the topical treatments podophyllotoxin and imiquimod are most commonly used for external genital warts, and in some cases destructive treatment and/or surgical removal is also necessary. Internal genital warts on the other hand usually require destructive treatment and/or surgical removal [19]. All of these treatment options are associated with some degree of discomfort, skin/mucosal irritation, pain, and sometimes scarring.

Cervical cancer screening does not prevent HPV infection, but two prophylactic HPV vaccines were licensed in Europe in 2006 and 2007 respectively, with the hope of eliminating the HPV infections that cause cancerous lesions. In 2010, the Swedish National Board of Health and Welfare launched the quadrivalent HPV vaccination program, in which girls 10–12 years of age are vaccinated for free as part of the school vaccination program. The quadrivalent HPV vaccination program aimed to protect against cervical cancer, cervical intraepithelial neoplasia (CIN), vulvar dysplasia, and genital warts caused by the HPV types 16, 18, 6 and 11. In order to estimate the benefits of the quadrivalent HPV vaccination program, decision-makers need to know the prevention, management, and treatment costs that were associated with these HPV-related diseases before the program was introduced. In this way, costs obtained before and after the introduction of HPV vaccination can be compared. As of yet, no study has done this in Sweden. Therefore, this study aimed to estimate the prevention costs (cytological cervical cancer screening), management costs (colposcopy and biopsy following inadequate and abnormal cytological results), and treatment costs of CIN, cervical cancer, and genital warts from a societal perspective in Sweden in 2009, 1 year before the introduction of the quadrivalent HPV vaccination program.

## Materials and Methods

### Data sources

#### Prevention, management, and treatment of cervical intraepithelial neoplasia

Data on the number of adequate cytological results (i.e. results that could be determined, including normal or abnormal results) and inadequate cytological results (i.e. results that could not be determined) were obtained from a 2009 report on the Swedish cervical cancer screening program [18]. The report did not provide information on the management of inadequate or abnormal cytological results, nor on the number or distribution of treatment procedures for women with histologically confirmed CIN grade 2 or worse (CIN2+). Therefore, to calculate these costs we used the guidelines of the Swedish cervical cancer screening program (available in Swedish at http://www.sfog.se). As per the guidelines, it was assumed that women with inadequate cytological results were referred back to the outpatient clinic for repeat cytology performed by a midwife, and that women with abnormal cytological results were referred to a gynecologist for immediate colposcopy and biopsy. Also as per the guidelines, it was assumed that women with histologically confirmed CIN2+ had one follow-up visit to undergo loop electrode excision procedure (LEEP), which is the most common treatment currently utilized in Swedish health care. The number of treatment procedures performed among women with CIN2+ was taken from statistics presented by the National Board of Health and Welfare [13]. As per the guidelines, no treatment procedure was included for women with CIN1, as the recommendations state that further follow-up should be performed 12 months later to prevent possible overtreatment. Travel time to and from the care site, wait time, and procedure or treatment time were estimated from our previous studies (travel time to the care site: 44 min; wait time: 10 min; initial cytology visit: 13 min; follow-up visit: 30 min; treatment: 60 min) [20,21].

#### Treatment of cervical cancer

The number of incident and prevalent cases of cervical cancer and cervical cancer mortality in Sweden were retrieved from the NORDCAN Database [22]. To estimate the cost of prevalent cancer and avoid repeat inclusion of treatment, we assumed that the reported number of women diagnosed with cervical cancer before 2009 were treated the same year they were diagnosed, and included only the cost of follow-up procedures for these cases.

Women with histologically confirmed cervical cancer were assumed to be referred for clinical staging according to FIGO procedures, and treated as previously reported in detail [21]. For cases of terminal cervical cancer, it was assumed that these women were previously diagnosed with cervical cancer and died from the disease in 2009. A clinical expert on palliative care and data from published literature were consulted to estimate a palliative care procedure for the last 120 days of life [23,24]. Palliative care was assumed to be performed mainly at the patient’s home [25] and included visits by each paramedical profession: three visits by a physician, twenty visits by a specialized nurse, and two visits each by a nutrition specialist, an occupational therapist, a counselor, and a physiotherapist. The last 14 days of life were assumed to have taken place in hospice care.

#### Treatment of genital warts

There is a lack of register data on genital warts, as they are not included in Sweden’s mandatory surveillance of infectious diseases. Therefore, for individuals under 45 years of age we used epidemiological data from a recent study based on prescription drug registers and patient registers to estimate the incidence of genital warts in Sweden in 2009 [26]. In addition, due to lack of Swedish estimates, published data from the United Kingdom in 2009 were used to estimate the incidence of genital warts in people 45 years of age and older, as well as to estimate rates of recurrent genital warts (i.e. reappearance within 3 months of initial treatment) and rates of persistent genital warts (i.e. persistence that requires further treatment 3 months after initial treatment) for all age groups [27]. These estimates from the United Kingdom were deemed the best available to estimate the comprehensive burden of genital warts in Sweden, with respect to persistence, recurrence, and all-age epidemiology. Indeed, although a report from the European Center or Disease Control on sexually transmitted infections showed a wide variation in chlamydia rates across Europe, the rates from the United Kingdom and Sweden were similar [28]. Moreover, a report from the United Kingdom showed peaks (*rates* sic) in the number of new and recurrent cases of genital warts among 20–24-year-olds that were similar to case-reported data from Sweden [26,29]. Although there are treatment guidelines for genital warts in Sweden, the clinical application of these guidelines varies [19,30]. For this reason a standardized questionnaire (S1 File) was developed and sent to 10 physicians with extensive clinical experience in managing and treating patients with genital warts, several of whom are well-known clinical experts (clinical expert panel). Physicians in the clinical expert panel represented different specialties (general medicine, dermatology/venereology, and gynecology/obstetrics) from both outpatient and inpatient care sites. Following completion of the questionnaire, physicians in the clinical expert panel were interviewed to review their responses. Responses were then pooled, and the means, medians, and ranges were calculated and used to estimate treatment patterns, average number of visits, and time spent for each type of procedure. The mean rates were used to construct the treatment patterns, which then determined the costs. Estimates of travel time to and from care sites and wait time were taken from our previous study (travel to the care site: 44 min; wait time: 10 min) [20].

### Costs

A societal perspective, including all direct medical costs and indirect productivity-related costs, was used to estimate costs, which are presented in Euro (€) using the 2009 average exchange rate (€ 1 = Swedish Kroner 10.62) (Table 1). Direct costs were based on Patient- Level Clinical Costing, referred to in Sweden as the cost per patient (KPP), which is a method used to calculate the cost of a stay or visit at a care site for an individual, or a group of patients. From a diagnostic perspective, this method describes health care utilization and is useful for decision-making in the health care sector. Productivity losses due to work absences related to the prevention and treatment of the diseases and premature death due to the diseases were quantified as indirect costs. Productivity losses were quantified by the human capital approach, i.e. the period-related income of the population concerned [31]. Due to the lack of age-specific registry data, we used average values from the official statistics. Indirect costs included were related to travel time to and from the care site, wait time and treatment time. As a proxy for the value of a patients’ time, we used the national average monthly wage rate for 2009 (defined as income from employment, self-employment, pension, sick pay, and other taxable incomes) for both women and men aged 16–64 years, and added charges for social benefits of 31.42% (available in English at www.scb.se). Assuming 20 workdays per month and the activity rate in Sweden, the cost per work day (€138.4) and work hour (€22.9) was used to calculate indirect costs. Based on the cost per work day, total indirect costs were calculated for the prevention, management, and treatment of CIN, as well as the treatment of cervical cancer and genital warts. No indirect costs were estimated for women and men aged 65 years and older, or aged 16 years or younger.

**Table 1:** Unit cost of resources expressed in 2009 Euros (€).

Resources	Direct Cost	Indirect Cost^a)^	Total Cost
**Prevention, management, and treatment of CIN**			
Visit to midwife for Pap smear^b)^	65	26	91
Follow-up visit for colposcopy and biopsy^b)^	228	32	260
LEEP or other procedure^c)^	291	44	335
**Treatment of cervical cancer** ^d)^			
FIGO Ia1-Ib1	12 317	5 197	17 514
FIGO Ib2	34 177	9 773	43 950
FIGO II	35 353	9 773	45 126
FIGO III	31 352	9 773	41 125
FIGO IV	31 352	20 068	51 420
**Palliative care** ^e)^			
Visit by a paramedical profession	65		65
Cost of 1hospice stay of 24 h	590	138	728
Average cost for 1 day of care at home	111	138	249
Average cost for palliative care	22 305	16 607	38 912
**Genital warts**			
Initial visit to physician ^f)^	85	29	114
Destructive treatment ^f)^	160	32	192
Surgical excision ^f)^	734	32	766
Imiquimod 5% cream tube ^g)^	52		52
Podophyllotoxin 5mL ^g)^	17		17

^a)^ Indirect productivity-related costs are estimated based on travel time to and from care site, wait time and visit time [20], treatment, and follow-up or sick leave due to cervical cancer [21].

^b)^ KPP [32].

^c)^ KPP 2009.

^d)^ Total cost including staging, treatment, and follow up [21].

^e)^ Costs included procedure and pharmacological treatment at home and hospice care retrieved from the palliative care unit and advanced home care unit, Långbro Park, Stockholm County Council. Average cost per day of care at home was based on calculations from a previous Swedish report on advanced care at home in Sweden [25].

^f)^ KPP 2009, clinical expert panel.

^g)^ The Dental and Pharmaceutical Benefits Agency (available in English at http://www.tlv.se)

CIN: cervical intraepithelial neoplasia; LEEP: loop electrode excision procedure; KPP: cost per patient

#### Prevention, management, and treatment of cervical intraepithelial neoplasia

Direct and indirect costs (Table 1) were mainly obtained from our previous studies [20, 21, 32] and KPP was obtained from the Patient Level Clinical Costing database. Costs for cytological testing and follow-up with colposcopy and biopsy included laboratory costs, physician assessment of abnormal results, and test costs. Costs for LEEP and other procedures were average estimates of all treatment procedures derived from the Patient Level Clinical Costing database in 2009, and represent a mean cost per women treated with LEEP or other procedures. In the sensitivity analysis all direct costs were decreased by 25% to reflect possible outpatient costs.

#### Treatment of cervical cancer

The resources used to calculate the treatment costs associated with incident cervical cancer were based on our previous study [21]. These costs included diagnosis, staging, and full treatment of FIGO stage I-IV cancer, pharmaceuticals, in- and outpatient care, palliative care, and follow-up. Due to the lack of official data, the proportion of women treated for different FIGO stages of cancer was based on the empirical number of patients diagnosed and treated according to a care algorithm applied in Stockholm-Gotland in 2003–2005, which is presented in the aforementioned study [21]. Two sensitivity analyses were performed: 1) using 50% of the treatment costs for incident cervical cancer. For prevalent cervical cancer, the estimated number of follow-up procedures for women who were previously diagnosed with cervical cancer and were alive in 2009 was multiplied by the cost of a follow-up visit with a physician as previously reported in detail; 2) using ±25% of the cost of follow-up of prevalent cervical cancer cases.

For terminal cervical cancer, the number of patients with who died in 2009 was multiplied by all palliative care costs (Table 1), including procedural and pharmacological treatment at home [25] and hospice care retrieved from the palliative care unit at ASIH Långbro Park, Stockholm County Council (Dr M. Bäcklund, personal communication), which represents how most women with palliative care are managed in Sweden. Variation in treatment and follow-up procedures and palliative care does exist in actual practice. Therefore, a variation of ±10% and an estimation of only 60 days of care at home was used in the sensitivity analyses.

#### Genital warts

Direct costs of visits for non-pharmacological or pharmacological reasons, or for treatment including diathermy, cryotherapy, laser, or surgical excision, were extracted from the Patient Level Clinical Costing database. Costs for topical cream treatments were obtained from The Dental and Pharmaceutical Benefits Agency (available in English at http://www.tlv.se). Indirect costs included costs related to travel time, applied to both genders, and treatment time [20,21]. To calculate these indirect costs, travel time, visit time, and treatment time were multiplied by wage rate estimates. No indirect costs were estimated for patients less than 16 years of age, or 65 years of age or older. To obtain the overall costs of genital warts, we used the estimated percentage of people who had a related procedure or a visit to a physician, multiplied by the relevant number of cases of genital warts (i.e. incident, recurrent, and persistent). This number was then multiplied by the cost of the treatment or visit, including the cost of topical creams. In the sensitivity analysis, all direct costs, including diathermy, cryotherapy, and surgical excision, were decreased by 25% to reflect possible outpatient costs. To reflect the uncertainty in the number of unrecorded cases, we increased incident and recurrent rates by 25%.

#### Ethical issues

This study and the survey of the clinical expert panel were approved by the Ethical Review Board at Karolinska Institutet, Stockholm, Sweden. All participants in the clinical expert panel were contacted initially by phone or e-mail, and those who agreed to participate received a written description of the research project and a questionnaire by e-mail. Their return of the questionnaire was considered sufficient as informed consent, and the Ethical Review Board at Karolinska Institutet supported this decision. Experts were asked to use their own clinical expertise and experience within this field to complete the questionnaire. They were not asked to consult patient records prior to completion of the questionnaire and no patient information is given in the questionnaires.

## Results

### Prevention, management, and treatment of cervical intraepithelial neoplasia

The 2009 the Swedish cervical cancer screening program reported a total of 650 178 cytological tests, of which 611 617 (94%) revealed normal results, 6 502 (1%) inadequate results, and 32 509 (5%) abnormal results [18]. Of all colposcopy and biopsy referrals, 10 049 women were treated for CIN2+ by LEEP or other procedure, with one estimated follow-up procedure within the same year. The estimated total annual cost of prevention, management, and treatment of CIN was €74 million, €8.5 million of which consisted of costs for colposcopy and biopsy referrals and €6 million of treatment costs. In sensitivity analyses we lowered the direct medical costs for initial cytological tests, management of inadequate and abnormal findings, and treatment of CIN2+ by 25% and 50% to reflect possible alternative outpatient costs. This decreased the total cost of the prevention, management, and treatment of CIN to €60.1 and €46.3 million, respectively, the majority of which consisted of direct medical costs. Due to uncertainty in the number of women treated for CIN annually we doubled the number of treated women, which increased the total annual cost of the prevention, management, and treatment of CIN to €79.9 million.

### Treatment of cervical cancer

In 2009 there were 441 women with incident cervical cancer in Sweden, the majority (68%) of whom were under 65 years of age, and 158 women died from cervical cancer, the majority (63%) of whom were older than 65 years of age [22]. Applying an estimated percentage of women in each FIGO stage based on empirical data from the Swedish Cancer Registry resulted in 205 women with FIGO Ia1-Ib1, 45 with FIGO Ib2, 98 with FIGO IIa-IIb, 66 with FIGO IIIa-IIIb, and 27 with FIGO IVa-IVb) [33]. Applying the cost of staging and treatment to these incident cervical cancer cases resulted in an annual cost of €15.8 million (Table 2). There were a total of 9 651 women with prevalent cervical cancer, the majority (57%) of whom were below 65 years of age. Applying follow-up procedures according to recommended time intervals resulted in an annual cost of €2.7 million. A total cost of €23 million was estimated for management and treatment of new and prevalent cervical cancer cases and palliative care. In the sensitivity analysis we used only half of the cost of the new cases, half of the cost for prevalent cases, and only 60 days of palliative care. This decreased the cost for the treatment and management of cervical cancer and palliative care by €11.3 million.

**Table 2:** Annual cost of prevention, management, and treatment of CIN and cervical cancer in Sweden, expressed in 2009 Euros (€).

Items	N^a)^	Direct cost (€)	N^a)^	Indirect cost (€)	Total cost (€)
Normal cytological result	611 617	39 421 014	611 617	15 890 350	55 311 365
Abnormal cytological result	32 509	9 521 287	32 509	1 885 516	11 406 803
Inadequate cytological result	6 502	838 745	6 502	338 093	1 176 838
Management and treatment costs for CIN^b)^	10 049	5 219 453	10 049	763 724	5 983 177
**Prevention cost**		**55 000 500**		**18 877 683**	**73 878 183**
FIGOIa1-Ib1	205	3 446 326	140	1 050 713	4 497 039
FIGOIb2	45	1 698 328	31	434 289	2 132 617
FIGO IIa-IIb	98	3 810 702	67	945 848	4 756 550
FIGO IIIa-IIIb	66	2 308 148	45	635 812	2 943 960
FIGO IVa-IVb	27	957 037	19	542 801	1 499 838
**Treatment cost of incident cervical cancer**	441	**12 220 541**	300	**3 609 463**	**15 830 004**
**Management cost of prevalent cervical cancer**	9 651	**2 403 713**	5 481	**264 009**	**2 667 722**
**Palliative care cost**	158	**3 524 187**	90	**979 808**	**4 503 995**
**Total annual cost (€)**		**73 148 941**		**23 730 964**	**96 879 904**

N = number of women

^a)^ Cases were multiplied by the number of follow up-procedures according to recommendations after treatment as previously described in detail [21].

^b)^ Average cost of LEEP and other procedure included 1 follow-up procedure according to current national guidelines (available at http://www.sfog.se).

CIN: cervical intraepithelial neoplasia; LEEP: loop electrode excision procedure

### Treatment of genital warts

We applied the figures obtained by extrapolating data from the Swedish study [26] and data from the United Kingdom [27] to the Swedish population. This rendered an estimated 18 196 incident cases of genital warts (seeking or receiving treatment) and 10 548 cases of recurrent and persistent genital warts in Sweden (approximately 46% women and 54% men). Results from the clinical expert panel showed that an average of 87% patients had external genital warts only, 10% had both external and internal genital warts, and 3% had internal genital warts only. The treatment patterns and percentage of patients with external, external and internal, or internal genital warts did not differ by gender and are therefore presented together (Table 3). The majority of patients with incident external and external and internal genital warts received pharmacological treatment (i.e. topical creams), while the majority of patients with internal genital warts received the wait-and-see option. Overall, destructive treatment was more common among recurrent and persistent cases of genital warts. Estimated visit time in the absence of destructive or surgical treatment was 22 min; destructive or surgical treatment visit time was estimated at 31 min. For both incident and recurrent cases, the average number of visits needed for patients with pharmacological treatment, destructive treatment, combination treatment, or surgical treatment was estimated at: 2, 2.5, 2.3, and 1.6 respectively. The total cost of 28 744 cases of genital warts in 2009 was €9.8 million (Table 4). The total annual cost of treating external genital warts was estimated at €8.1 million, while costs for external and internal genital warts were estimated at €1.2 million, and internal genital warts alone at €0.5 million (S1, S2 and S3 Tables). Within the total cost, that proportion attributable to pharmacological treatment was estimated at €2.9 million, while that for destructive treatment was €3.4 million. Costs for surgical excision were €1.9 million and combination treatment €0.9 million. The total cost of the management and treatment of recurrent and persistent cases was €4.5 million. In the sensitivity analyses we lowered the direct costs for a visit to a physician and treatment by 25% to reflect possible outpatient costs. This decreased the total cost of the treatment of genital warts to €7.9 million.

**Table 3:** Number and mean percentage of patients undergoing different treatment procedures for genital warts based on estimates from the clinical expert panel.

	External		External and Internal		Internal	
	n = 25 008, (87%)		n = 2 874, (10%)		n = 862, (3%)	
Treatment option	Incident	Recurrent	Incident	Recurrent	Incident	Recurrent
	n,(%)	n,(%)	n,(%)	n,(%)	n,(%)	n,(%)
**Wait and see**	**5 224, (33)**	**918, (10)**	**400, (22)**	**63, (6)**	**232, (42.5)**	**76, (24)**
**Pharmacological treatment**	**7 282, (46)**	**247, (27)**	**800, (44)**	**127, (12)**	**8, (1.5)**	**5, (1.5)**
Podophyllotoxin	6 627, (91)	1 338, (54)	777, (97)	103, (81)	8, (100)	5, (100)
Imiquimod	655, (9)	1 140, (46)	24, (3)	24, (19)	0, (0)	0, (0)
**Destructive treatment**	**2 533, (16)**	**3 487, (38)**	**454, (25)**	**432, (41)**	**104, (19)**	**66, (21)**
Cryotherapy	583, (23)	732, (21)	123, (27)	151, (35)	0, (0)	0, (0)
Diatermy	1 241, (49)	1 360, (39)	255, (56)	177, (41)	26, (25)	17, (25)
Laser	735, (29)	1 395, (40)	77, (17)	104, (24)	78, (75)	50, (75)
**Combination treatment**	**317, (2)**	**1 560, (17)**	**18, (1)**	**190, (18)**	**0, (0)**	**5, (1.5)**
Destructive treatment and podophyllotoxin	317, (100)	1 466, (94)	18, (100)	169, (89)	0, (0)	5, (100)
Destructive treatment andimiquimod	0, (0)	94, (6)	0, (0)	21, (11)	0, (0)	0, (0)
**Surgical excision**	**475, (3)**	**734, (8)**	**146, (8)**	**243, (23)**	**202, (37)**	**165, (52)**
**Total n, (%)**	**15 831, (100)**	**9 177, (100)**	**1 819, (100)**	**1 055, (100)**	**546, (100)**	**316, (100)**

**Table 4:** Cost of treating genital warts in Sweden, expressed in 2009 Euros (€).

Treatment option	Incident		Recurrent		
	Direct cost	Indirect cost	Direct cost	Indirect cost	Total cost
**Wait and see**	**497 793**	**160 121**	**89 837**	**28 897**	**776 648**
**Pharmacological treatment**	**1 652 314**	**442 428**	**615 031**	**142 669**	**2 852 441**
Podophyllotoxin	1 513 566	405 276	295 148	79 030	2 293 020
Imiquimod	138 748	37 151	319 883	63 640	559 421
**Destructive treatment**	**1 246 732**	**238 274**	**1 594 436**	**304 727**	**3 384 168**
Cryotherapy	282 154	53 925	353 468	67 554	757 101
Diatermy	608 714	116 337	621 568	118 793	1 465 413
Laser	355 863	68 012	619 400	118 379	1 161 653
**Combination**	**13 638**	**23 548**	**724 066**	**123 408**	**884 661**
Destructive treatment and podophyllotoxin	13 638	23 548	668 129	115 356	820 671
Destructive treatment and imiquimod	0	0	55 938	8 052	63 990
**Surgical excision**	**965 896**	**40 240**	**804 201**	**55 839**	**1 866 177**
**Total**	**4 376 372**	**904 609**	**3 827 571**	**655 540**	**9 764 094**

### Total annual cost

The estimated total annual cost associated with the prevention, management, and treatment of CIN, cervical cancer, and genital warts in 2009 was €106.6 million, of which €81.4 million (76%) were direct medical costs. Depending on the sensitivity analysis, total annual cost ranged from €66 million to €121 million.

## Discussion

Overall, the results showed that in 2009 the total annual costs associated with the prevention, management, and treatment of CIN, cervical cancer, and genital warts were considerable, at €107 million. Of these costs, €74 million (70%) were associated with the prevention, management, and treatment of CIN, and an additional €23 million (20%) with the treatment of incident and prevalent cervical cancer, which occurs despite the existence of the Swedish cervical cancer screening program, and palliative care. The estimated annual cost of the treatment of incident and prevalent cervical cancer cases is close to the estimated cost of prophylactic HPV vaccination in Sweden [34]. The cost of genital warts for both men and women was €9.8 million, over half of which was related to incident cases. In future generations, HPV vaccination has the potential to substantially reduce the costs associated with the prevention, management, and treatment of HPV-related diseases.

To our knowledge, this is the first attempt to estimate the annual costs of the HPV-related diseases cervical dysplasia, invasive cervical cancer, and genital warts in Sweden. The estimates are based on the best available data, i.e. the Swedish cervical cancer screening program, epidemiological data on the incidence and prevalence of cervical cancer, and mean estimates of managing and treating genital warts obtained from clinical experts via self-administered questionnaires and reviewed responses.

However, this study has several limitations that need to be acknowledged. For example, our overall treatment costs for genital warts were similar to those from other studies [30,35], but exact data on clinical management is lacking. Therefore, estimates for the clinical management of genital warts were derived from a clinical expert panel, consisting of well-known physicians from various parts of Sweden with expert knowledge on the treatment of genital warts. However, the clinical practices of the physicians in the expert panel may not be representative of all physicians in Sweden, which may lead to bias. Also, the clinical expert panel was not asked to check their patient records. Instead they were asked to answer based on their experience, which may have led them to overestimate or underestimate in their responses. In addition, the estimated number of recurrent and persistent cases of genital warts in Sweden was calculated based on epidemiological data from the United Kingdom since clinical data on this subject has yet to be published in Sweden. Nevertheless, a majority of cases of genital warts in Sweden are treated pharmacologically with podophyllotoxin, and an estimated 50% recurrence rate in baseline cases is probably an accurate assumption [36,37]. Incidence data is likely underestimated, as it was not possible to ascertain the number of cases from primary care not receiving pharmacological treatment [26]. Moreover, vaccine-related reductions in genital warts likely started as early as 2008 in Sweden, as this is when the vaccine was subsidized for girls aged 13–17 years, in addition to the fact that the vaccine was available on-demand starting in late 2007.

There are also limitations concerning screening and the number of treatment procedures for women with histologically confirmed CIN. Women who were diagnosed with a CIN1 lesion before 2009 that later developed into CIN2+ would have been referred to immediate treatment, which was not accounted for. Also, in 2009 some county councils used liquid-based cytology and HPV testing to follow-up abnormal cytological results, which may have led to an overestimation of costs. Moreover, women who are HPV-negative at this follow-up are not referred for colposcopy or biopsy, thus the total number of referrals in our study would be somewhat lower.

Travel time to and from care sites was based on estimates from Stockholm, which may not be representative of all of Sweden, and costs may therefore be underestimated for geographical areas where distances are greater.

We believe that the assumptions made to calculate costs are valid, given the existence of national guidelines for the HPV-related diseases considered in this report, and surveillance data for screening. However, there is a possible underestimation of costs associated with incident and prevalent cervical cancer as potential overlaps in the use of health care resources for incident and prevalent cases diagnosed during the previous year cannot be determined. No costs for recurrent cases were accounted for, which would likely underestimate the total annual costs for the treatment of prevalent cervical cancer cases. Our cost estimates for the treatment of cervical cancer by FIGO stage were similar to those used in a study from the United Kingdom [38].

Our study highlights the significant economic burden (€107 million) of the HPV-related diseases cervical dysplasia, invasive cervical cancer, and genital warts for the 9.34 million affected inhabitants of Sweden in 2009, which is somewhat higher than other studies performed in European countries. A study from the United Kingdom reported annual direct costs for cervical cancer screening, management, and treatment of genital warts of £208 million (for 60 million inhabitants) [39]. In France, annual overall direct cost of cervical cancer screening, management of abnormal tests, and treatment of CIN was estimated at €336 million (for 60 million inhabitants) [40]. A separate study estimated the additional annual direct cost associated with the management of diagnosed invasive cervical cancer in France at almost €44 million [41]. In Belgium, estimated annual cost from a societal perspective for screening was €65 million, with an additional €16 million for the management of CIN, cervical cancer, and genital warts (for 10.5 million inhabitants) [2]. The differences between countries could possibly be explained by differences in their cervical cancer screening programs, such as start age for screening, frequency, time intervals, number of follow-up visits, and cut-offs for referral to immediate treatment, which may influence the difference in direct medical costs. Also, in the base-case scenario, our direct medical costs for an initial visit for cytological testing reflected costs for a hospital visit to a midwife. The study from the United Kingdom [39] reported a lower direct medical cost of an initial visit for cytological testing compared to our study. Using half of the direct medical cost of an initial visit and colposcopy referrals decreased the total annual direct medical costs to €25 million, i.e. similar to results in aforementioned studies. Overall, parameter variations within primary screening have a significant impact on overall costs associated with the prevention of cervical HPV-related diseases. However, the direct medical costs for an outpatient visit for screening needs to be evaluated to confirm the actual cost and plausible ranges. One aim of future research should be to update Swedish cost data on outpatient costs for preventive health care services to reduce the uncertainty associated with the present estimates. Moreover, costs related to complications after treatment for precancerous and cancerous lesions are difficult to estimate, but it is likely that they are not negligible, e.g. obstetrical and perinatal complications after conization [42].

We used the human capital approach to estimate the value of productivity losses due to work absenteeism related to cervical cancer and genital warts. One critique that is often raised with this approach is that it discriminates against elderly who are no longer part of the workforce. This is of course highly relevant for the indirect costs for cervical cancer, since the majority of women diagnosed are of retirement age. However, the screening program affects women of working age, making the indirect costs higher for this particular group. In order to obtain a more accurate deliberation of all indirect costs imposed on Swedish society as a result of cervical cancer and genital warts, a comprehensive assessment is necessary and must be carried out using national registry-based data, which provide individual characteristics including age at diagnosis, disease severity, and inability to work due to sick leave, early retirement, or premature death, which would contribute to heterogeneity in our study population and might influence the total estimated indirect cost.

This cost analysis gives important insight into HPV-related diseases in Sweden since it provides recent cost data on the prevention, management, and treatment of CIN, cervical cancer, and genital warts from a societal perspective. Despite the limitations of this study, these results are of interest due to the recent introduction of the quadrivalent HPV vaccination (against HPV6, 11, 16, and 18) program. In clinical trials, the quadrivalent vaccine has been shown to reduce lifetime risk of cervical cancer caused by the included HPV types by 47% to 100%, depending on age and coverage [43,44], and to reduce the risk of genital warts by 83% [45]. As Sweden has kept its cervical cancer screening program unchanged and added quadrivalent HPV vaccination, a reduction in the future economic burden of management and treatment costs for CIN, cervical cancer, and genital warts is expected. Previous cost-effectiveness studies have shown that adding a quadrivalent HPV vaccine to an existing cervical cancer screening program is cost-effective [46–48]. The World Health Organization-funded PRIME modelling study concluded that in 156 of 179 countries, prophylactic HPV vaccination in girls is cost-effective [48]. Implementation of organized prophylactic HPV vaccination will hopefully lead to a situation where a greater portion of resources are spent on prevention and a decreasing portion on manifest disease management.

There are still questions as to whether boys should be included in the HPV vaccination program, and how to wisely design the Swedish cervical cancer screening program to adapt to a situation with a lower incidence and prevalence of precancerous lesions. The costs reported in this study may be incorporated into future cost-effectiveness analyses comparing strategies with different available HPV vaccines alongside the cervical cancer screening program.

This study only provides a partial estimate of the cost of HPV-related diseases. Other HPV-related diseases such as cancer of the vulva, vagina, anus, penis, head and neck, and recurrent respiratory papillomatosis were not included in this analysis. Future research should aim to estimate the total direct medical and indirect costs associated with all major HPV-related diseases in Sweden, and to identify cost drivers of relevance to guide decision-making regarding future investments in HPV prevention programs.

## Supporting Information

S1 File Questionnaire. (DOCX)

S1 Table Cost of treating *external* genital warts in Sweden, expressed in 2009 Euro (€). (DOCX)

S2 Table Cost of treating *external and internal* genital warts in Sweden, expressed in 2009 Euro (€). (DOCX)

S3 Table Cost of treating *internal* genital warts in Sweden, expressed in 2009 Euro (€). (DOCX)
