# Supplementary material for: Cost of Preventing, Managing, and Treating Human Papillomavirus (HPV)-Related Diseases in Sweden before the Introduction of Quadrivalent HPV Vaccination
Source: PLoS One. 2015 Sep 23;10(9):e0139062. doi: 10.1371/journal.pone.0139062 (PMC4580320; doi:10.1371/journal.pone.0139062)
Supplement: S1 File — (DOCX) [file pone.0139062.s001.docx]

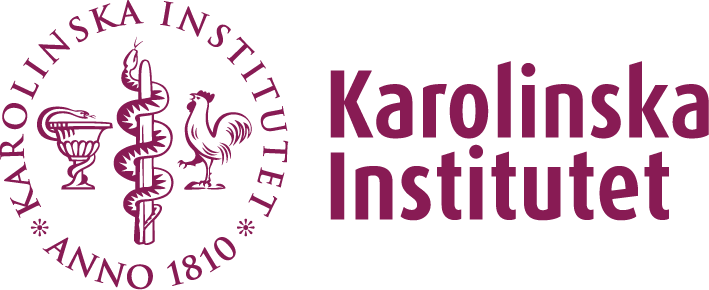


**S1 File. Questionnaire**

**Expert survey on management procedures and treatment pathways for females and males with ICD-10-CM code A63 ´condyloma acuminatum´ as the primary diagnose.**

**Part 1. Responder characteristics**

|  |  |
| --- | --- |
| **1.Q1** | **My main clinical specialty is:**  1. General medicine  2. Dermatology/venereology  3. Gynecology/Obstetrics  4. Other……………………………………………………………………………………………..  ……………………………………………………………………………………………………………….. |
| **1.Q2** | **My main experience of patients seeking medical care due to condyloma is from:**  1. Primary care youth clinics  2. Dermatology/venereology outpatient clinic  3. Dermatology/venereology at hospital  4. Gynecology/Obstetrics outpatient clinic  5. Gynecology/Obstetrics at hospital  6. Other…………………………………………………………………………………………….. |

**Part 2. Patient characteristics and initial management**

|  |  |
| --- | --- |
| **2.Q1** | **Among your patients seeking medical care because of anogenital condyloma, please estimate the percentage of men and women.**  Men _____ %  Women _____ %  **Sum: 100 %** |
| **2.Q2** | **Among female patients, please estimate the percentage of women presenting with:**  External condylomata exclusively ______%  External and internal condylomata ______%  Internal condylomata exclusively ______%  **Sum: 100%** |
| **2.Q3** | **Among male patients, please estimate the percentage of women presenting with:**  External condylomata exclusively ______%  External and internal condylomata ______%  Internal condylomata exclusively ______%  **Sum: 100%** |
| **2.Q4A** | **Among female patients, please estimate the proportion of patients that you refer to be managed by another clinical specialist than your own (e.g. dermatology/venereological clinics, gynecologist, surgeon, urologist, infectious medicine)** _____ % (0-100%) |
| **2.Q4B** | **Among female patients with condyloma that you refer to another clinic or a physician with another specialty than your own, please estimate the proportion to which the main reason of referral was:**  **A:** The location ______ %  **B :** Large areas affected ______ %  **C:** Atypical appearance ______ %  **D:** Recurrence after one or more treatment  cycles regardless of chosen treatment _______%  **E:** Resistance to given therapy _______%  **F:** Other, such as: _________________ _______%  **Sum: 100%**  Comments:___________________________________________________________________________________________________________________________________________________________________________________________________________________________ |
| **2.Q5A** | **Among male patients, please estimate the proportion of patients that you refer to be managed by another clinical specialty than your own (e.g. dermatology/venereological clinics, gynecologist, surgeon, urologist, infectious medicine)** _____ % (0-100%) |
| **2.Q5B** | **Among male patients with condyloma that you refer to another clinic or a physician with another specialty than your own, please estimate proportion to which the main reason of referral was:**  **A:** The location ______ %  **B :** Large areas affected ______ %  **C:** Atypical appearance ______ %  **D:** Recurrence after one or more treatment  cycles regardless of chosen treatment _______%  **E:** Resistance to given therapy _______%  **F:** Other, such as: _________________ _______%  **Sum: 100%**  Comments:___________________________________________________________________________________________________________________________________________________________________________________________________________________________ |
| **2.Q6A** | **Please estimate the percentage of female patient for which you need further diagnostic evaluation (0-100% for A-E each). Only your own interventions should be included in estimations.**   1. Biopsy of skin/external mucosa_____% 2. HPV genotyping _____% 3. Colposcopy _____% 4. Anoscopy _____% 5. Other, such as _______________________________________ ______% |
| **2.Q6B** | **Please estimate the percentage of male patient for which you need further diagnostic evaluation (0-100% for A-E each). Only your own interventions should be included in estimations.**   1. Biopsy of skin/external mucosa_____% 2. HPV genotyping _____% 3. Anoscopy _____% 4. Other, such as _______________________________________ ______% |

**Part 3. Treatment practices**

**Example: This is an example on how to assess the estimated percentage for treatment patterns of patients with external anogenital warts. Please assess the estimated percentage for treatment patterns in question: Q 3.1A, Q3.2A and Q3.3A (NB: the vertical percentages should add up to 100%):**

| **Treatment option** | **Incident cases (%)** |  | **Recurrent cases (%)** |  | **Persistent cases (%)** |  |
| --- | --- | --- | --- | --- | --- | --- |
|  | **Women** | **Men** | **Women** | **Men** | **Women** | **Men** |
| **A) Wait and see** | **20** | **10** | **30** | **20** | **40** | **30** |
| **B)Pharmacological treatment, of which:** | ***80*** | ***90*** | ***70*** | ***80*** | ***60*** | ***70*** |
| *Podophyllotoxin* | *70* | *80* | *50* | *70* | *40* | *60* |
| *Imiquimod* | 10 | 10 | 20 | 10 | 20 | 10 |
| *Other (please add information*):* | *0* |  |  |  |  |  |
| **C) Destructive treatment, of which:** | ***0*** | ***0*** | ***0*** | ***0*** | ***0*** | ***0*** |
| *Cryotherapy* |  |  |  |  |  |  |
| *Diatermy* |  |  |  |  |  |  |
| *Laser* |  |  |  |  |  |  |
| *Other (please add information**):* |  |  |  |  |  |  |
| **D)Combination procedure, of which:** | ***0*** | ***0*** | ***0*** | ***0*** | ***0*** | ***0*** |
| *Destructive treatment and podophyllotoxin* |  |  |  |  |  |  |
| *Destructive treatment and imiquimod* |  |  |  |  |  |  |
| *Other (please add information***):* |  |  |  |  |  |  |
| **E)Surgical excision** | *0* | *0* | *0* | *0* | *0* | *0* |
| **Total** | **100%** | **100%** | **100%** | **100%** | **100%** | **100%** |

*Alternative pharmacological treatment: ______________________________________________________

**Alternative destructive treatment strategy: ____________________________________________________________________________________________________________________________________________________________________

***Alternative combination procedure strategy: ____________________________________________________________________________________________________________________________________________________________________

**3.Question 1 A. Please assess the estimated percentage for treatment patterns of patients with external condyloma, according to your own clinical experience:**

Not applicable (if not applicable, for example if you do not manage this category of patients at your clinic, please proceed to Question 2.)

| **Treatment option** | **Incident cases (%)** |  | **Recurrent cases (%)** |  | **Persistent cases (%)** |  |
| --- | --- | --- | --- | --- | --- | --- |
|  | **Women** | **Men** | **Women** | **Men** | **Women** | **Men** |
| **A) Wait and see** |  |  |  |  |  |  |
| **B)Pharmacological treatment, of which:** |  |  |  |  |  |  |
| *Podophyllotoxin* |  |  |  |  |  |  |
| *Imiquimod* |  |  |  |  |  |  |
| *Other (please add information*):* |  |  |  |  |  |  |
| **C) Destructive treatment of which:** |  |  |  |  |  |  |
| *Cryotherapy* |  |  |  |  |  |  |
| *Diatermy* |  |  |  |  |  |  |
| *Laser* |  |  |  |  |  |  |
| *Other (please add information**):* |  |  |  |  |  |  |
| **D)Combination procedure, of which:** |  |  |  |  |  |  |
| *Destructive treatment and podophyllotoxin* |  |  |  |  |  |  |
| *Destructive treatment and imiquimod* |  |  |  |  |  |  |
| *Other (please add information***):* |  |  |  |  |  |  |
| **E)Surgical excision** |  |  |  |  |  |  |
| **Total** | **100%** | **100%** | **100%** | **100%** | **100%** | **100%** |

*Alternative pharmacological treatment: ______________________________________________________

**Alternative destructive treatment strategy: ____________________________________________________________________________________________________________________________________________________________________

***Alternative combination procedure strategy: ____________________________________________________________________________________________________________________________________________________________________

|  |  |
| --- | --- |
| **3.Q1B** | **Please estimate the average time allocated (including documentation) for a clinic visit of a patient with external condyloma, given that no destructive or surgical treatment is carried out during the visit.**  ___________ minutes  Comments:  ______________________________________________________________________________________________________________________________________________________ |
| **3.Q1C** | **Please estimate the average time allocated for a patient with external condyloma (including documentation), given that destructive or surgical treatment is carried out during the visit.**  ___________ minutes  Comments:  ___________________________________________________________________________ |
| **3.Q1D** | **Please estimate the average number of clinic visits needed for a patient with external condyloma , within a calendar year from first treatment with:**  Pharmacological treatment: ______  Destructive treatment: ______  Combination procedure: ______  Surgical excision: ______  Comments:  ______________________________________________________________________________________________________________________________________________________ |

**3.Question 2 A. Please assess the estimated percentage for treatment patterns of patients with external and internal condyloma, according to your own clinical experience:**

Not applicable (if not applicable, for example if you do not manage this category of patients at your clinic, please proceed to Question 3.)

| **Treatment option** | **Incident cases (%)** |  | **Recurrent cases (%)** |  | **Persistent cases (%)** |  |
| --- | --- | --- | --- | --- | --- | --- |
|  | **Women** | **Men** | **Women** | **Men** | **Women** | **Men** |
| **A) Wait and see** |  |  |  |  |  |  |
| **B)Pharmacological treatment, of which:** |  |  |  |  |  |  |
| *Podophyllotoxin* |  |  |  |  |  |  |
| *Imiquimod* |  |  |  |  |  |  |
| *Other (please add information*):* |  |  |  |  |  |  |
| **C) Destructive treatment of which:** |  |  |  |  |  |  |
| *Cryotherapy* |  |  |  |  |  |  |
| *Diatermy* |  |  |  |  |  |  |
| *Laser* |  |  |  |  |  |  |
| *Other (please add information**):* |  |  |  |  |  |  |
| **D)Combination procedure, of which:** |  |  |  |  |  |  |
| *Destructive treatment and podophyllotoxin* |  |  |  |  |  |  |
| *Destructive treatment and imiquimod* |  |  |  |  |  |  |
| *Other (please add information***):* |  |  |  |  |  |  |
| **E)Surgical excision** |  |  |  |  |  |  |
| **Total** | **100%** | **100%** | **100%** | **100%** | **100%** | **100%** |

*Alternative pharmacological treatment: ______________________________________________________

**Alternative destructive treatment strategy: ____________________________________________________________________________________________________________________________________________________________________

***Alternative combination procedure strategy: ____________________________________________________________________________________________________________________________________________________________________

|  |  |
| --- | --- |
| **3.Q2B** | **Please estimate the average time allocated (including documentation) for a clinic visit of a patient with external and internal condyloma, given that no destructive or surgical treatment is carried out during the visit.**  ___________ minutes  Comments:  ______________________________________________________________________________________________________________________________________________________ |
| **3.Q2C** | **Please estimate the average time allocated for a patient with external and internal condyloma (including documentation), given that destructive or surgical treatment is carried out during the visit.**  ___________ minutes  Comments:  ______________________________________________________________________________________________________________________________________________________ |
| **3.Q2D** | **Please estimate the average number of clinic visits needed for a patient with external and internal condyloma, within a calendar year from first treatment with:**  Pharmacological treatment: ______  Destructive treatment: ______  Combination procedure: ______  Surgical excision: ______  Comments:  ______________________________________________________________________________________________________________________________________________________ |

**3.Question 3 A. Please assess the estimated percentage for treatment patterns of patients with internal condyloma, according to your own clinical experience:**

Not applicable (if not applicable, for example if you do not manage this category of patients at your clinic.)

| **Treatment option** | **Incident cases (%)** |  | **Recurrent cases (%)** |  | **Persistent cases (%)** |  |
| --- | --- | --- | --- | --- | --- | --- |
|  | **Women** | **Men** | **Women** | **Men** | **Women** | **Men** |
| **A) Wait and see** |  |  |  |  |  |  |
| **B)Pharmacological treatment, of which:** |  |  |  |  |  |  |
| *Podophyllotoxin* |  |  |  |  |  |  |
| *Imiquimod* |  |  |  |  |  |  |
| *Other (please add information*):* |  |  |  |  |  |  |
| **C) Destructive treatment of which:** |  |  |  |  |  |  |
| *Cryotherapy* |  |  |  |  |  |  |
| *Diatermy* |  |  |  |  |  |  |
| *Laser* |  |  |  |  |  |  |
| *Other (please add information**):* |  |  |  |  |  |  |
| **D)Combination procedure, of which:** |  |  |  |  |  |  |
| *Destructive treatment and podophyllotoxin* |  |  |  |  |  |  |
| *Destructive treatment and imiquimod* |  |  |  |  |  |  |
| *Other (please add information***):* |  |  |  |  |  |  |
| **E)Surgical excision** |  |  |  |  |  |  |
| **Total** | **100%** | **100%** | **100%** | **100%** | **100%** | **100%** |

*Alternative pharmacological treatment: ______________________________________________________

**Alternative destructive treatment strategy: ____________________________________________________________________________________________________________________________________________________________________

***Alternative combination procedure strategy: ____________________________________________________________________________________________________________________________________________________________________

|  |  |
| --- | --- |
| **3.Q3B** | **Please estimate the average time allocated (including documentation) for a clinic visit of a patient with internal condyloma, given that no destructive or surgical treatment is carried out during the visit.**  ___________ minutes  Comments:  ______________________________________________________________________________________________________________________________________________________ |
| **3.Q3C** | **Please estimate the average time allocated for a patient with internal condyloma (including documentation), given that destructive or surgical treatment is carried out during the visit.**  ___________ minutes  Comments:  ______________________________________________________________________________________________________________________________________________________ |
| **3.Q3D** | **Please estimate the average number of clinic visits needed for a patient with internal condyloma, within a calendar year from first treatment with :**  Pharmacological treatment: ______  Destructive treatment: ______  Combination procedure: ______  Surgical excision: ______  Comments:  ______________________________________________________________________________________________________________________________________________________ |
