# Supplementary material for: Cost of Preventing, Managing, and Treating Human Papillomavirus (HPV)-Related Diseases in Sweden before the Introduction of Quadrivalent HPV Vaccination
Source: PLoS One. 2015 Sep 23;10(9):e0139062. doi: 10.1371/journal.pone.0139062 (PMC4580320; doi:10.1371/journal.pone.0139062)
Supplement: S1 Table — (DOCX) [file pone.0139062.s002.docx]

**S1 Table. Cost of treating *external* genital warts in Sweden, expressed in 2009 Euro (€)**

|  | Incident |  | Recurrent |  |  |
| --- | --- | --- | --- | --- | --- |
| Treatment option | Direct cost | Indirect cost* | Direct cost | Indirect cost* | Total |
| **Wait and see** | **444 046** | **142 833** | **78 002** | **25 090** | **689 971** |
| **Pharmacological treatment** | **1 487 138** | **398 200** | **586 513** | **135 488** | **2 607 339** |
| Podophyllotoxin | 1 353 296 | 362 362 | 273 241 | 73 164 | 2 062 061 |
| Imiquimod | 133 842 | 35 838 | 313 273 | 62 325 | 545 278 |
| **Destructive treatment** | **1 023 285** | **195 569** | **1 394 868** | **266 585** | **2 880 307** |
| Cryotherapy | 233 025 | 44 535 | 292 922 | 55 983 | 626 466 |
| Diathermy | 496 445 | 94 880 | 543 998 | 103 968 | 1 239 292 |
| Laser | 293 814 | 56 153 | 557 947 | 106 634 | 1 014 549 |
| **Combination treatment** | **12 897** | **22 268** | **643 095** | **109 721** | **787 981** |
| Destructive treatment and podophyllotoxin | 12 897 | 22 268 | 597 361 | 103 138 | 700 499 |
| Destructive treatment and imiquimod |  |  | 45 733 | 6 583 | 52 317 |
| **Surgical excision** | **557 741** | **23 236** | **517 305** | **35 919** | **1 134 201** |
| **Total (€)** | **3 525 107** | **782 105** | **3 219 783** | **572 804** | **8 099 798** |
