# Supplementary material for: Cost of Preventing, Managing, and Treating Human Papillomavirus (HPV)-Related Diseases in Sweden before the Introduction of Quadrivalent HPV Vaccination
Source: PLoS One. 2015 Sep 23;10(9):e0139062. doi: 10.1371/journal.pone.0139062 (PMC4580320; doi:10.1371/journal.pone.0139062)
Supplement: S2 Table — (DOCX) [file pone.0139062.s003.docx]

**S2 Table. Cost of treating *external and internal* genital warts in Sweden, expressed in 2009 Euro (€)**

|  | **Incident** |  | **Recurrent** |  |  |
| --- | --- | --- | --- | --- | --- |
| **Treatment option** | **Direct cost** | **Indirect cost*** | **Direct cost** | **Indirect cost*** | **Total** |
| **Wait and see** | **34 027** | **10 945** | **5 379** | **1 730** | **52 081** |
| **Pharmacological treatment** | **163 503** | **43 780** | **27 548** | **6 921** | **241 753** |
| Podophyllotoxin | 158 598 | 42 467 | 20 938 | 5 606 | 227 609 |
| Imiquimod | 4 905 | 1 313 | 6 610 | 1 315 | 14 144 |
| **Destructive treatment** | **181 960** | **34 776** | **172 987** | **33 061** | **422 784** |
| Cryotherapy | 49 129 | 9 390 | 60 546 | 11 571 | 130 636 |
| Diathermy | 101 898 | 19 475 | 70 925 | 13 555 | 205 852 |
| Laser | 30 933 | 5 912 | 41 517 | 7 935 | 86 297 |
| **Combination treatment** | **741** | **1 280** | **79 038** | **13 353** | **94 413** |
| Destructive treatment and podophyllotoxin | 741 | 1 280 | 68 834 | 11 885 | 80 719 |
| Destructive treatment and imiquimod |  |  | 10 204 | 1 469 | 11 673 |
| **Surgical excision** | **170 955** | **7 122** | **170 948** | **11 870** | **360 895** |
| **Total (€)** | **551 186** | **97 903** | **455 902** | **66 936** | **1 171 927** |
