# Supplementary material for: Cost of Preventing, Managing, and Treating Human Papillomavirus (HPV)-Related Diseases in Sweden before the Introduction of Quadrivalent HPV Vaccination
Source: PLoS One. 2015 Sep 23;10(9):e0139062. doi: 10.1371/journal.pone.0139062 (PMC4580320; doi:10.1371/journal.pone.0139062)
Supplement: S3 Table — (DOCX) [file pone.0139062.s004.docx]

**S3 Table. Cost of treating *internal* genital warts in Sweden, expressed in 2009 Euro (€)**

|  | **Incident** |  | **Recurrent** |  |  |
| --- | --- | --- | --- | --- | --- |
| **Treatment option** | **Direct cost** | **Indirect cost*** | **Direct cost** | **Indirect cost*** | **Total** |
| **Wait and see** | **19 720** | **6 343** | **6 455** | **2 076** | **34 595** |
| **Pharmacological treatment** | **1 672** | **448** | **969** | **260** | **3 349** |
| Podophyllotoxin | 1 672 | 448 | 969 | 260 | 3 349 |
| Imiquimod | 0 | 0 | 0 | 0 |  |
| **Destructive treatment** | **41 487** | **7 929** | **26 581** | **5 080** | **81 077** |
| Cryotherapy | 0 | 0 | 0 | 0 | 0 |
| Diathermy | 10 372 | 1 982 | 6 645 | 1 270 | 20 269 |
| Laser | 31 115 | 5 947 | 19 936 | 3 810 | 60 808 |
| **Combination treatment** | **0** | **0** | **1 934** | **334** | **2 267** |
| Destructive treatment and podophyllotoxin | 0 | 0 | 1 934 | 334 | 2 267 |
| Destructive treatment and imiquimod |  |  | 0 | 0 |  |
| **Surgical excision** | **237 200** | **9 882** | **115 948** | **8 051** | **371 081** |
| **Total (€)** | **300 079** | **24 602** | **151 887** | **15 801** | **492 369** |
